# The role of the tumor microenvironment in drug resistance acquisition in lung squamous cell carcinoma

**DOI:** 10.1186/s13046-026-03682-x

**Published:** 2026-03-04

**Authors:** Oana Zanoaga, Cornelia Braicu, Andreea Nutu, Ioana Berindan-Neagoe, Andreas Bender

**Affiliations:** 1 Department of Genomics, MEDFUTURE Institute for Biomedical Research, Iuliu Hațieganu University of Medicine and Pharmacy, Cluj-Napoca, Romania; 2 Doctoral School, “Iuliu Haţieganu” University of Medicine and Pharmacy, 23 Marinescu, Cluj-Napoca, 400337 Romania; 3 Romanian Academy of Medical Sciences, Bucharest, Romania; 4 Center for Biotechnology, College of Medicine and Health Sciences, Khalifa University of Science and Technology, Abu Dhabi, United Arab Emirates; 5 Centre for Molecular Informatics, Department of Chemistry, University of Cambridge, Lensfield Road, Cambridge, CB2 1EW UK; 6 STAR-UBB Institute, Babeş-Bolyai University, Cluj-Napoca, Romania

**Keywords:** Tumor microenvironment, Lung squamous cell carcinoma, Drug resistance

## Abstract

Lung squamous cell carcinoma (LUSC), a subtype of non-small cell lung cancer, exhibits significant therapeutic challenges, among others, due to the lack of known driver mutations as well as the development of drug resistance. In LUSC, the extracellular matrix (ECM), closely linked to dynamic changes in the tumor microenvironment (TME), plays a key role in regulating tumor immunity, through complex interactions among these components. These interactions drive the emergence of resistance mechanisms, including hypoxia-induced adaptive responses, immune evasion, and ECM and TME remodeling, which collectively contribute to reduced treatment efficacy and tumor persistence. Furthermore, cancer-associated fibroblasts and tumor-associated macrophages promote the proliferation and survival of tumor cells by forming protective barriers around them. Understanding the complex crosstalk between LUSC cells and their microenvironment is crucial for developing novel therapeutic strategies that aim to overcome drug resistance. This review highlights the latest findings on the role of the TME in therapy resistance and discusses potential targets for improving treatment outcomes in this cancer type.

## Introduction

Lung cancer represents one of the most prevalent and lethal malignancies worldwide and is broadly categorized into non-small cell lung cancer (NSCLC), which comprises approximately 85% of all cases- including lung adenocarcinoma (LUAD) and lung squamous cell carcinoma (LUSC), the two main NSCLC subtypes, and small cell lung cancer (SCLC), accounting for about 15% of cases [1].

LUSC originates from the epithelial cells lining the central bronchi. It accounts for approximately 25–30% of all NSCLC cases and is typically characterized by the presence of keratinization, intercellular bridges, and distinct histopathological features [2, 3]. It is strongly linked to lifestyle factors, including smoking, environmental factors like exposure to biofuels, and underlying respiratory conditions like chronic obstructive pulmonary disease [4].

LUSC poses significant treatment challenges due to its frequent diagnosis at advanced stages and is associated with poor prognosis [5]. Despite extensive research, effective targeted therapies for LUSC remain limited [1, 6–8]. Furthermore, LUSC often shows inactivation of tumor suppressor genes such as TP53, KEAP1, PTEN, and CDKN2A, with few directly targetable driver mutations [9–11]. Therefore, their translation into effective targeted therapies remains limited, leaving chemotherapy (docetaxel, gemcitabine and platinum-based chemotherapy) and immunotherapy (nivolumab, pembrolizumab, atezolizumab, cemiplimab, ipilimumab) as the main treatment options [5, 12, 13]. The urgent need to develop new therapeutic strategies is also related to the 5-year survival rates that remains below 18% for LUSC patients [14, 15], and below 20% for LUAD patients [16–18], with most patients developing resistance to therapy.

The tumor microenvironment (TME) is a dynamic and complex network consisting mainly of different cellular components, ECM, and signaling molecules (including cytokines and growth factors) [19]. It plays a pivotal role in cancer progression and metastasis by providing complex interactions between immune cells and cancer cells [20, 21]. The effectiveness of existing treatments is further hampered by the high heterogeneity of the TME, which varies widely among patients [22]. This heterogeneity significantly influences disease progression and response to therapy, particularly in the context of immunotherapy [22–24]. In lung cancer, the TME significantly affects the efficacy of drug therapies, such as immune checkpoint inhibitors (ICI) (e.g., nivolumab and pembrolizumab targeting PD-1/PD-L1, and ipilimumab targeting CTLA-4), cancer vaccines, and adoptive cell therapies like CAR-T cells [8]. Characterization of immune infiltration landscape revealed that LUSC exhibit a higher immune infiltration that include T-cells and macrophages [25]. Moreover, LUSC patients with immune inflammation displayed higher immune checkpoint marker expression [26]. Genomic alterations in LUSC influence tumor development in a subtype-specific manner, permitting the identification of the optimal immunotherapy options [27], such as a higher tumor mutational burden being associated with increased sensitivity to immunotherapy of LUSC tumors [28].

Overall, both intrinsic cellular mechanisms and extrinsic TME factors are involved in LUSC disease recurrence and development of drug resistance. This review gives an overview of ECM remodeling, stroma cell-mediated signaling, and immune suppression as central mechanisms driving therapeutic resistance. By highlighting molecular aspects of the LUSC microenvironment, how interactions among the ECM, immune cells, stromal components, and tumor cells influence drug efficacy and identify potential targets for improved therapeutic strategies.

### Tumor-TME interplay in LUSC

**Fig. 1:**
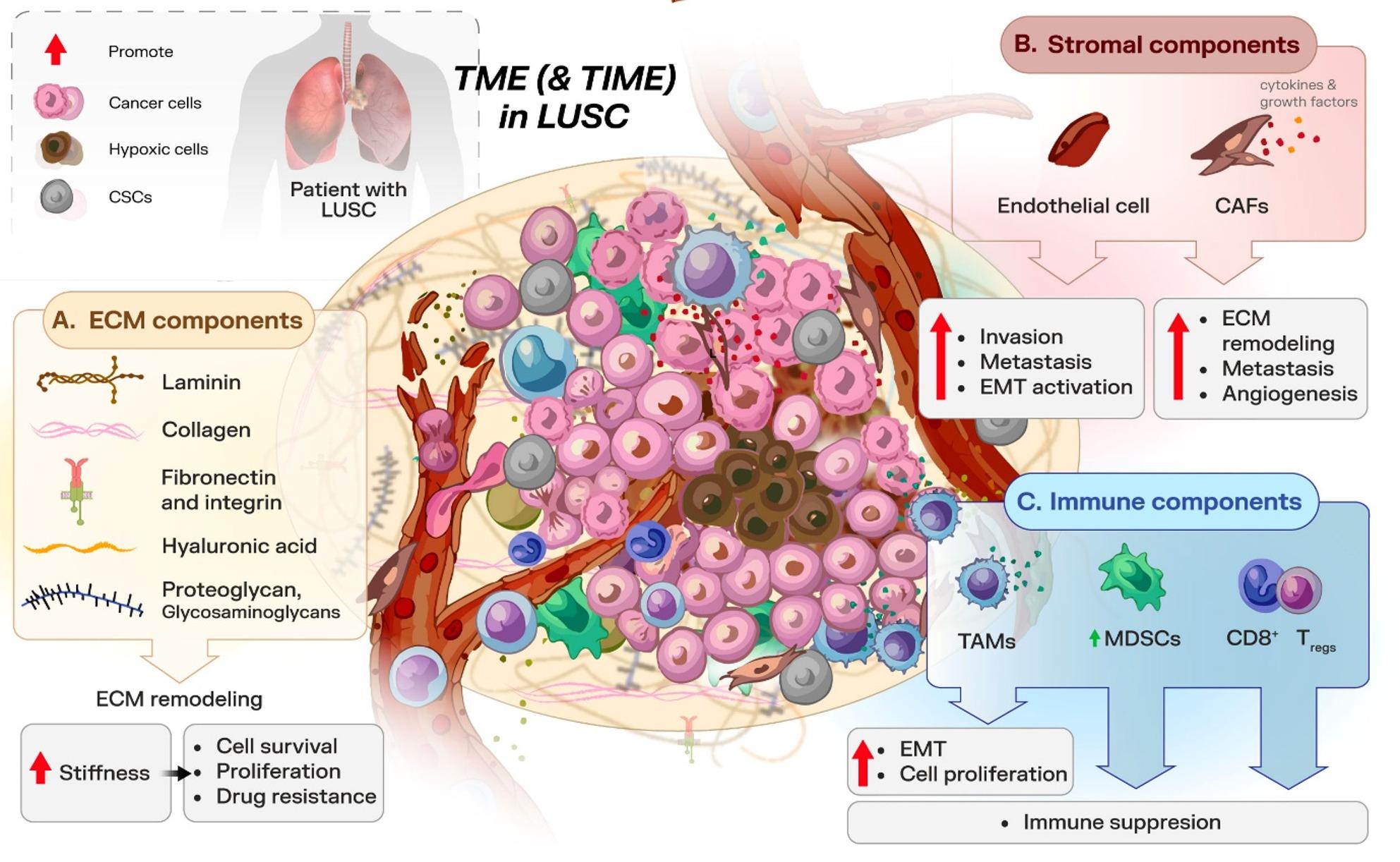
Overview of LUSC-TME key components and interactions: immune cells, ECM, and stromal. **A** The ECM components provide structural support and signaling that influence tumor behavior. **B** Stromal components like CAFs and endothelial cells contribute to the tumor's vascularization and extracellular structure, aiding tumor progression and survival. **C** Immune components include TAMs, MDSCs, and T cells, which interact with tumor cells, often promoting tumor growth or suppressing immune responses. Hypoxia further drives angiogenesis, ECM remodeling, and immunosuppressive signaling, creating a supportive niche that facilitates tumor progression and therapy resistance. This environment highlights the complexity of the TME involvement in LUSC and the significant challenges associated with developing effective therapeutic strategies

The interplay between the TME in LUSC is a dynamic and complex process that deeply influences tumor biology, disease progression, and treatment outcomes. LUSC is characterized by a complex TME composed of immune cells, fibroblasts, endothelial cells, and ECM components that interact dynamically with tumor cells [29], see Fig. 1. Each cellular component interacts with the microenvironment through paracrine and autocrine signaling via cytokines and mediators or through direct or indirect cell-cell interactions, driving tumor growth, invasion, and metastasis [30–32]. Table 1 summarizes the main TME components and their respective roles in LUSC.

**Table 1:** The main components of the TME and their specific role in LUSC

**TME component**	**Effects on TME**	**Mechanisms and targets in LUSC**
ECM	Cellular heterogeneity [24]; physical and chemical barrier to drug penetration [33]; interact with signaling molecules (e.g., TGFβ, IL6,TNFα [34]	pro-tumorigenic environment [24]; matrix stiffening promotes a tumor vasculature [35]; regulate immunological surveillance [36], drug resistance [37], interacts with integrins and growth factor receptors (e.g. α_v_β_3_ integrin interact with the TGFβIIR) [38]; prognosis role [39, 40]
CAFs	Supportive microenvironment to tumor development and resistance to therapy [41, 42]; secrete cytokines and growth factors (IL6, FGF) [43, 44]	Promote ECM remodeling and metastasis [41, 42]; upregulation of m6A modification of COL10A1 increasing reactive oxygen species (ROS) generation [42, 45]; activate pro-survival signaling pathways (e.g., STAT3, MAPK) [42, 46, 47]
Endothelial cells	Aggressive behavior, promoting invasion and metastasis [48], interact with secrete cytokines and growth factors [49, 50]	Promote invasion, metastasis and EMT activation [51]; hypoxia-induced HIF1α signaling [52]; regulate tumor plasticity [48]
Immune cells	Dual role promoting and inhibiting tumor growth [53]; secrete cytokines (IL1β, IL6, IL8, IL10, TGF-β, TNF-α, VEGF, CXCL12) [54, 55]	Tregs, and CD8+ T cells regulate immune evasion [56]; neutrophils modulate inflammation and PD-L1 mediates immunosuppression [57]; regulate angiogenesis and EMT, enhancing metastasis [49]
CSCs	Subpopulation of tumor cells responsible capable for self-renewal and tumor initiation, driving tumorigenesis and therapy resistance[58, 59] via cytokines and growth factors (IL-6) [60]	Subpopulation of tumor cells, contribute to tumor recurrence and metastasis [58, 59]; regulate Notch, Wnt/β-catenin and Hedgehog promote pro-tumorigenic activities [61, 62]; prognostic and therapeutic target [63]

In LUSC, tumor cells can develop various immunosuppressive mechanisms that allow them to evade immune surveillance and promote survival and proliferation after treatment [64]. Understanding these TME-driven mechanisms is essential for targeting immune escape and therapeutic resistance in LUSC.

LUSC tumors secrete immunosuppressive cytokines (e.g., TGFβ, IL10) that alter the TME, reducing antitumor immune responses and promoting resistance to immunotherapy and chemotherapy [65, 66]. Furthermore, chronic hypoxia within the LUSC TME can induce metabolic reprogramming that further impairs T-cell function and enhances tumor cell adaptation to treatment [67]. Uppregulation of immune checkpoint molecules such as PD-L1, which inhibits T-cell activation has been associated with the recruitment of immunosuppressive cells like regulatory T cells (Tregs) and tumor-associated macrophages (TAMs) [68, 69].

Structural and compositional alterations in the tumor-associated ECM of LUSC generate a dense and mechanically stiff microenvironment that facilitates CAF-mediated EMT (epithelial-mesenchymal transition) and promotes tumor progression through the previously described signaling pathways [70]. Cancer-associated fibroblasts (CAFs) remodel the matrix architecture within the tumor stroma by aligning ECM components into parallel fibers, thereby facilitating cancer cell migration and invasion [71]. This structural reorganization of the ECM not only promotes tumor cell motility but also influences endothelial cell behavior, supporting angiogenesis and the formation of abnormal, leaky vasculature [35]. Lu et al. exhibited in an interesting review that through reciprocal signaling with endothelial cells, CAFs help establish a pro-invasive microenvironment that sustains tumor growth, enhances vascular permeability, and enables intravasation of cancer cells into the bloodstream [72]. The two TME clusters in LUSC have been identified in an recent bioinformatic study based on the relative abundance of 24 immune cells with distinct immune features. Moreover, the study demonstrated that the immune infiltration pattern may serve as a prognostic predictor in LUSC, and a five marker combination (HCST, VAMP5, NAPSB, SOD2, EVI2A) was developed to accurately distinguishing TME clusters [73].

Cancer stem cells (CSCs) are distinct subpopulation of tumor cells characterized by their capacity for self-renewal, with important role in tumorgenesis and tumor progression [63]. Their resistance to chemotherapy and other therapeutic approaches is strongly influenced by complex interactions with the TME, which provides survival signals and protection under stress conditions. In addition, CSCs can disseminate to distant sites and generate supportive niches that promote metastatic colonization and tumor expansion, underscoring their contribution to cancer aggressiveness and treatment failure [58, 74–76]. This supportive niche was confirmed in a recent study conducted both in vitro and in vivo. In this study, integrin β4 (ITGB4) and stemness-associated factor SOX2 were found overexpressed and correlated with cisplatin resistance for CSCs isolated from LUSC patients [59]. Furthermore, Jiang et al. developed and validated an eight-gene CSCs prognostic signature (PPP1R27, TLX2, ANKLE1, TIGD3, AMH, KCNK3, FLRT3, and PPBP) in LUSC [63]. This study highlighted the key role of the crosstalk between CSCs and TME in prognosis of LUSC patients.

Together, these interactions establish a resilient and adaptive tumor ecosystem, underscoring the need for therapeutic strategies that target both the tumor and its surrounding microenvironment. These differences are not only descriptive, but can have prognostic and therapeutic implications. Furthermore, integrating multi-marker predictive models enables more accurate classification of TME subtypes, offering opportunities to refine patient stratification and guide personalized therapy. Thus, the tumor-TME interplay represents an important determinant in LUSC pathogenesis and serves as a foundation for advancing immunotherapy and precision oncology approaches.

### TME and smoking

Smoking, the principal etiologic factor for LUSC [77, 78], not only increases carcinogenic mutational burden but also shapes the TME in distinct manner. In LUSC, a higher tobacco-associated mutational signature correlates with increased tumor mutational cargo, immune cell infiltration, cytolytic activity, and interferon-γ signaling, indicative of a more inflamed TME in smokers compared with non-smokers [79]. Integrative analyses from The Cancer Genome Atlas (TCGA) further show that smoking alters immune cell composition, including changes in activated and resting NK cells and endothelial cells, contributing to immune dysfunction within the TME [80]. Additionally, smoking intensity is associated with increased PD-L1 expression and higher infiltration of CD4⁺ and CD8⁺ T cells and macrophages in NSCLC, including LUSC, suggesting that tobacco exposure modulates both immune checkpoints and cellular infiltrates that influence antitumor immunity [79, 81]. Collectively, these findings indicate that smoking profoundly affects TME composition and function in LUSC, with important implications for immunotherapy and TME-oriented combination therapy strategies [79].

### TME altered transcriptomic patterns and their role in LUSC prognosis and therapeutic response

TME is increasingly recognized as a critical determinant of disease progression, prognosis, and therapeutic response in LUSC. Multiple TME-related molecular and cellular signatures have been identified, reflecting the complex interplay between tumor cells, stromal components, and ECM. Immune-related gene signatures, including 13-gene immune response panels [82], follicular helper cell activity [83], and immune regulatory clusters [84, 85], have shown strong associations with patient outcomes and responses to immunotherapy. Likewise, stromal or ECM-based signatures [24, 85, 86], CAF markers [87], and adhesion genes [86], underscore the prognostic role of the tumor stroma in shaping tumor aggressiveness and therapy resistance. In addition, TME-linked biological processes, including hypoxia [88], EMT [88], cell death mechanism [86, 89] or fatty acid metabolism [90], contribute to tumor evolution and may provide predictive biomarkers for therapeutic efficacy. Collectively, these TME-derived signatures offer valuable tools for risk stratification and that will permit to develop novel personalized treatment strategies in LUSC (Table 2).

**Table 2:** Genetic signatures in the TME and their role in LUSC prognosis and therapeutic response

Key biology affected by signature	Genes	Type of data used	Key Findings	Reference
13-gene immune response signature	↑KLRC2, CD1E, LIM2, NPY, CDH12, OTX2, ADRA1D, FGL1, ZFP42, GAGE2A, FGF4, F13A1, SAMD9L	504 samples from TCGA and 606 samples from GSE30219, GSE12472, GSE157011 and GSE78220	Prognostic- immunotherapy response	[91]
ECM matrix risk signature	↑RSPO1, CTHRC1, SPP1, MMRN1, COL10A1, and PRG4	223 LUSC patients from GSE201221, GSE50081, GSE108124, GSE165192, TCGA databases	Predictors of premalignant progression	[24]
Immunological and ECM signature	↑ADAM12, MMP1, SERPINE1, PLOD3, and P4HA3	TCGA databases GSE17536, GSE71187, GSE78229, Human Protein Atlas	Prognostic biomarkers directly related to ECM protein dynamics	[85, 86]
Immune signature	↑CD47, CD73, SIRPA, and TIM-3	440 LUSC and 49 normal tissue samples LUSC-TCGA database	Prediction and stratification of stage I–III LUSC patients	[84, 85]
Immune signature	↑FGA and CSF2	489 LUSC tissues and 49 normal tissues fromTCGA database	prognostic signature	[92]
Immune signature	↑LAPTM5, C1QC, CSF1R and SLCO2B1	GSE21933, GSE33479, GSE33532, GSE62113, GSE74706	Regulate tumor infiltration of immune cells Influence patient survival outcomes	[93]
TME cluster	↑HCST, VAMP5, NAPSB, SOD2, EVI2A	TCGA data base, GSE3141, GSE8894, GSE19188, GSE29013, GSE30219, GSE37745, GSE43580, GSE50081, and GSE115457	2 TME clusters based on the relative abundance of 24 immune cells; immune infiltration pattern could be a prognosis predictor in LUSC	[73]
Stromal and immune signature	↑BHMT2, FES, HSPB7, NOVA2, LPAP2, and SEMA3B	TCGA database	Correlation between the TME and prognosis in LUSC	[94]
CAF signature	↑CLDN1, TMX4, ALPL, PTX3, BHLHE40, TNFRSF12A, VKORC1, CST3 and ADD3	473 samples from TCGA-LUSCdataset	CAFs signature was used for multivariate Cox analysis- predicting the prognosis and immunotherapeutic response	[87]
Immune response follicular helper cells	↑ALKBH5, METTL3, HNRNPC and KIAA1429	122 tumor and 27 normal control samples from TCGA database, GSE67061, and GSE2088	sensitivity to immunotherapy and chemotherapy	[83]
Hypoxia and EMT	↑FSTL3, TNFRSF12A, PTP4A3, ILK, and SNAI1	GSE157009, GSE157010,TCGA databases	Predict immunotherapeutic response and prognosis	[88]
CSCs	P1R27, TLX2, ANKLE1, TIGD3, AMH, KCNK3, FLRT3, and PPBP	TCGA-LUSCdataset GSE30219, GSE37745	stemness-related prognostic model	[63]
Focal adhesion-10-gene prognostic model	↑ITGA3, VAV2, FLNC, FLT4, HGF, MYL2, ITGB1, PDGFRA, CCND2, and PPP1CB	TCGA databases	A good predictor of response to chemotherapy	[86]
Cell-cell death-associated signature related to TME	↑ATP6V0D1, ATP6V1B1, DRAM2, GPSM1, LRRK2, MAPK3, PINK1, RRAGB, AKT2, CIDEC, HTRA2, PTGIS, STK24, BAG4, CASP4, TNFRSF12A, TNFRSF8, TRADD	493 LUSC tissue and 49 adjacent normal tissue samples from TCGA cohort	Prognostic signature; predict response to immunotherapy	[89]
Pyroptosis regulatory patterns in the immune microenvironment	↑ NLRP3, GSDMD, NLRP1, CASP1, CASP3, TP53, and CASP5	502 tumor tissue samples and 49 normal tissue samples from LUSC-TCGA databases	Predict immunotherapy efficiency	[82]
Ferroptosis-related genes	↑ CP, CAV1, ATF3, HELLS, PLIN2, TFRC, RRM2, MUC1, ARRDC3, ACSL5, DUSP1, JUN, EPAS1, ROS1, MAP1LC3C, ENPP2, ALOX5, TP63, SLC39A8, SLC7A5	502 LUSC tissue samples and 49 normal samples fromTCGA databases	Prognostic signature	[95]
Fatty acid metabolism	↑ACOT11, APOH, BMX, CYP2R1, DPEP3, FABP6, FADS2, GLYATL2, and THR	396 patients from TCGA databases- early stages LUSC	Prediction of the prognosis of early-stage LUSC	[90]

↑: upregulation

### ECM receptor interaction

A PanCancer analysis of RNA expression from 43 collagen genes in TCGA solid tumors, including LUSC, revealed that collagen composition alone can classify tissue of origin and correlates with survival, immune landscapes, somatic mutations, and chromosomal alterations. For example, the presence of high levels of COL1A1 and and related fibrillar collagens in the tumors have been linked to shorter survival [39]. Using collagen expression, a machine learning classifier accurately predicted aneuploidy and chromosome arm CNA status across various cancers, underscoring a significant link between collagen ECM and tumor molecular changes. These insights could enhance our understanding of tumor ecosystems, offering new prognostic tools and therapeutic targets [48].

Analysis of gene expression proffiling pre- and post-radiotherapy showed activation of key pathways, including the ECM-receptor signaling pathway, with consistent upregulation of fibronectin 1 (FN1) and thrombospondin 1 (THBS1). Survival analysis identified FN1 and THBS1 as significant prognostic markers, highlighting their potential as predictive biomarkers for radiotherapy response in LUSC [40]. Another bioinformatic study revealed that CD168 and OPN (Osteopontin) genes were significantly enriched in the ECM-receptor interaction pathway, interacting with the receptor for hyaluronic acid-mediated motility (RHAMM). These genes have potential application as therapeutic targets for LUSC patients who tolerate immunotherapy [96].

### Adhesion molecules

Later, a novel focal adhesion-related gene signature (ITGA3, VAV2, FLNC, FLT4, HGF, MYL2, ITGB1, PDGFRA, CCND2, and PPP1CB) was identified for distinguishing and predicting the prognosis of LUSC, offering new insights into its diagnosis and treatment [86]. Taken together, these advances demonstrate that integrating ferroptosis-, hypoxia-, EMT-, and adhesion-related gene signatures contributes to a more comprehensive molecular understanding of LUSC, positioning multi-gene risk models as valuable tools for precision oncology and therapeutic decision-making.

### Cell death pathways

In LUSC, there is a significant correlation between the cell death mechanisms (which include mechanisms such as apoptosis and ferroptosis) and the tumor’s TME and stemness characteristics [82, 89, 97]. A recent study identified a cell death-associated signature (CDI, cell death index) in LUSC, which closely correlates with prognosis and TME [89]. This CDI may help predict patient prognosis and response to immunotherapy in LUSC; the genes that exhibited differential expression between the high- and low-risk groups included cytokines linked to cell death and were significantly associated with immune-related pathways [89]. Additionally, the high-risk group showed an increased presence of naive CD4 + T cells, monocytes, activated dendritic cells, and neutrophils, while plasma cells and resting memory CD4 + T cells were less abundant [89]. 

Pyroptosis, a form of programmed cell death associated with inflammation, impacts the TIME in LUSC. Patients with lower pyroptosis scores have shown better immune responses to ICI treatments [82]. By examining pyroptosis regulatory patterns within the TME, it is possible to predict clinical outcomes and responses to immunotherapy in LUSC patients [82].

A recent study has linked ferroptosis, a regulated form of cell death characterized by iron-dependent lipid peroxidation, with the immune status of tumors in LUSC. High-risk groups for LUSC exhibit a gene signature associated with ferroptosis, which correlates with the abundance of various immune cells [97]. Notably, this includes naive B cells, CD8⁺ T cells, activated memory CD4⁺ T cells, follicular helper T cells, and M1 macrophages. These immune cells play critical roles within the TME, shaping anti-tumor immune responses and influencing the efficacy of therapeutic interventions. Overall, these findings highlight the emerging role of ferroptosis as a critical link between tumor cell death mechanisms and immune regulation in LUSC, suggesting that targeting ferroptosis-related pathways could improve immunotherapy outcomes.

### Hypoxia

Zhuang et al. identified a six-gene risk score model (FSTL3, TNFRSF12A, PTP4A3, ILK, SNAI1, and SERPINE1) associated with hypoxia and EMT-related genes, which was validated in LUSC. This study underscores the prognostic significance of hypoxia- and EMT-driven molecular alterations, providing biomarkers that may refine patient stratification and risk assessment in LUSC [86].

### ECM components as therapeutic targets in LUSC

**Fig. 2:**
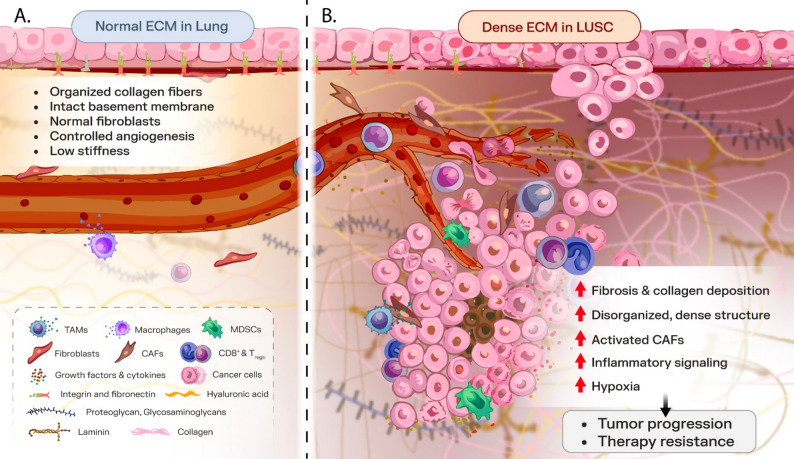
Schematic representation comparing the ECM structure in normal lung tissue versus lung cancer. **A** The normal ECM is organized and less rigid, allowing for controlled cell adhesion, migration, and signaling. **B** In contrast, the ECM in LUSC becomes denser and more disorganized, with excessive deposition of collagen and fibronectin, increased cross-linking, and altered biochemical properties. These changes enhance mechanical stiffness and create a pro-tumorigenic microenvironment that supports cancer progression. Notably, CAFs play a crucial role in remodeling the ECM, facilitating EMT and contributing to tumor invasiveness and therapy resistance

The ECM is a complex structural and biochemical network of proteins and glycoproteins, acting as a physical and chemical barrier, modulating cellular interactions, and creating a pro-tumorigenic environment [72]. Its architecture (shown schematically in Fig. 2) includes proteins such as collagen, fibronectin, laminin, glycosaminoglycans, hyaluronic acid, and proteoglycans, forming a dynamic scaffold for cellular interactions [7, 67, 73]. The ECM-driven characteristics are often linked to immune evasion, increased metastatic potential, and poor prognosis in LUSC, highlighting the dual role of the TME as both a mediator of immune regulation and a key contributor to therapeutic failure [72].

ECM act as a physical barrier by restraining drug penetration, and reducing treatment efficacy, while activating signaling pathways that enhance tumor survival [29]. Tissue architecture alteration and interstitial pressure stimulation are mechanisms that also inhibited effective drug delivery [24]; Table 3 provides an overview on related preclinical studies.

**Table 3:** Recent studies linking proteins from the ECM to modulating cellular interactions of relevance in LUSC

Type of study	ECM component	Model	Observation	Reference
Preclinical studies	↑Integrin α3 and α4	Calu-1 cell line	Diagnostic/prognostic markers; therapeutic targets	[98]
	↑PCDHA3	H520 cells	Therapeutic target, regulate EMT	[99]
	↑Collagen and TNC protein	Female BALB/c mice	Promote carcinogenesis and therapeutic targets	[100]
Clinical tudies	↑Fibronectin 1 (FN1) and thrombin reactive protein 1 (THBS1)	GSE162945, GSE162945	Predicting tumor response to radiotherapy	[40]
	↑Type III collagen alpha 1 (COL3A1)	GSE21656	Prognosis and cisplatin resistance	[101]
	↑43 collagen related genes	TCCA data sets	Predict therapy response	[39]
	↑ED-B fibronectin	13 tumor tissue samples	Target for antiangiogenic therapy	[102]
	↑MMP12	125 tissue samples	Diagnosis, treatment, poor outcomes	[103]
	↑TGM2	492 tumor and 49 normal samples	Associated with poor prognosis; affects tumor metastasis and chemotherapy resistance	[104]

↑: upregulation

ECM components interact with cell surface receptors, initiating intracellular signaling pathways that promote signal transduction and regulate cell survival, proliferation and drug resistance [74]. For example, fibronectin binding to integrins can activate the PI3K/AKT pathway, leading to increased resistance to apoptosis [75]. Also, ECM interactions with integrins can activate FAK signaling, further promoting cancer cell survival and resistance to therapy [76]. The ECM is critical in recruiting immune cells by activating PI3K/AKT, enhancing cancer stemness immune evasion [58].

Related to LUSC, degradation and posttranslational modifications of ECM proteins have been described [42]. A recent study found that protocadherin alpha 3 (PCHDA3) counteracts EMT by repressing mesenchymal markers (N-cadherin, fibronectin, and vimentin) and upregulating epithelial markers (E-cadherin and α-catenin). Moreover, PCDHA3 suppressed LUSC cell proliferation, migration and invasion by regulating EMT pathway [99].

A growing body of research underscores the important role of integrins as mediators of tumor–ECM interactions. The role of integrins in was demonstrated in a study that used RAGE (Rapid Amplification of Gene Expression) as a novel technique to evaluate transcriptomic alteration. Data from this study, validated by flow cytometry and immunohistochemistry, identified integrin α3 and α4 as being expressed in LUSC cells and potentially as molecular targets for therapy [98].

Preclinical studies using LUSC models have provided critical insights into the role of ECM remodeling in tumor progression and therapy response.Increased tissue rigidity is a specific feature of solid tumors that promotes cancer progression [105]. According to Zakaria et al., therehere is a correlation between tissue rigidity and the overexpression of collagen and tenascin-C (TNC) protein, and. Thus, targeting this factors can be considered a promising strategy in LUSC treatment [100]. For example, Integrin binding peptide LXY30 has the capacity to target PDX models of LUSC and to detect α3β1 integrin on the surface of live cancer cells. Furthermore, LXY30 can be used for in vivo targeted delivery of cancer drugs in NSCLC [106]. Accumulating data from various studies identified matrix metalloproteinases (MMPs) as enzymes that can irreversibly degrade complex substrates in the ECM [107–109], and are also involved in drug resistance and tumor immunity by activating innate immune responses [110]. ED-B fibronectin was found to be upregulated and has the potential to be used as antiangiogenic therapy, as demonstrated by Khan et al. in a study on 13 samples of LUSC and 15 samples of LUAD tissue. Here ED-B fibronectin from the tumor compartments and vascular endothelium was associated with tumor microvessel density [102], constituting another plausible mechanism for targeting LUSC.

### CAFs and drug response in LUSC

CAFs are a specific type of fibroblast and the most common component of the tumor stroma, which has been identified in the tumor mesenchyme of multiple cancers, including LUSC [42, 87]. CAFs play a multifaceted role in LUSC, contributing to ECM remodeling, therapeutic resistance, and immune modulation [111, 112], as summarized in Table 4. Notably, Notch [113], MAPK [47], and PI3K/AKT pathways [114] form complex regulatory networks within the TME, activated by CAFs [115]. The failure of targeted therapies can often be attributed to the activation of compensatory signaling pathways when a specific pathway in CAFs is inhibited, ultimately sustaining tumor progression and therapy resistance [114].

**Table 4:** TIME cellular components and association with mechanistic insight into cancer progression and drug response in LUSC

Cellular component	Markers	Data used, experiment type	Observation	Reference
CAF	↑COL10A1	Microarray, GSE22874 , 43 LUSC samples	Regulate cell proliferation and repress apoptosis-induced oxidative stress via METTL3 Mediated m^6^A Methylation of COL10A1	[42]
	↑CLDN1, TMX4, ALPL, PTX3, BHLHE40, TNFRSF12A, VKORC1, CST3, ADD3	scRNA-seq data (GSE153935, Drop-seq) 473 LUSC samples	Provides CAF prognostic signature for identifying potential prognostic CAFRGs and predicting the prognosis and immunotherapeutic response for LUSC	[87]
	↑miR-369	miRCURY LNA™ Universal RT microRNA PCR Human panel in co-culture system, paired tissue samples from 52 patients	Exosomal CAFs miR-369 potentiates migration and invasion via NF1-mediated MAPK signaling pathway	[47]
	↑SOX2	Microarray, Co-culture system	CAFs Suppress SOX2-induced dysplasia in co-culture.	[116]
	↑CD34 and CD248	Single-cell imaging mass cytometry (IMC) analysis, 401 LUSC samples	Fibroblast phenotypes-prognostic roles; were identified 11 CAF phenotypes	[117]
T cells	↓GADD45B	TIMER database	Enhances inflammation and normal immunity therapeutic target for anti-LC immunotherapy	[118]
	↑PD-1, CTLA4	Multiplex immunohistochemistry /27 LUSC samples	Immunotherapy response related to CD8 + T cell infiltration	[119]
	↑PD-L1, CTLA-4, IDO1, PD-L2, TIM-3, LAG-3 and TIGIT	scRNA-seq, 502 tumor tissue samples and 49 normal tissue samples from TCGA database	Markers predict prognosis and therapeutic response	[120]
TAMs	↓E-cadherin, ↑vimentin	Western blotting, ELISA, H226 and EBC-1 cells	Increases metastatic potential and tumor cell proliferation	[121]
NK cells	↑CD57 (TINK)	Immunohistochemistry, 50 tumor samples	CD57 expression may reflect functional exhaustion of NK cells, which could contribute to a poorer prognosis	[122]
	↑CD8	scRNA-seq, tumor and adjacent normal tissues from 9 LUSC patients	CD8 + cytotoxic T cells, NK cells, and distinct macrophage subsets, including immunosuppressive SPP1-expressing macrophage, influencing immune responses and tumor progression	[123]
Dendritic cells	↑GPC3	In vitro functional test, NCI-H226 and SK-MES-1 cell lines	Promote the growth, migration, and invasion of LUSC cells	[124]
MDSc cells	↑CD33+	Imaging Mass Cytometry, 12 tumor samples	Marker confers immune suppression	[125]
	↑ CD11b^+^	flow cytometry, FVB/N and C57BL/6 mice	CD11b+ cells in tumors promote immunosuppression by inducing ROS-generating M-MDSCs	[126]

↑:upregulation; ↓: downregulation

CAFs have an influence on most immune cell populations, generating an immunosuppressive TME that contributes to the development of resistance to immunotherapy [127–130]. Furthermore, a multitude of inflammatory factors are secreted by CAFs, activating various protumorigenic pathways, such as the SDF-1-CXCR4-dependent proliferation of CSCs [131]. Phenotypic and spatial features of CAFs in NSCLC have been identified through the correlation between CAF types and chemoresistance that showed the presence of SMA (smooth muscle actin) CAFs for LUSC patients who had received neoadjuvant chemotherapy [42, 132]. The patients have been classified into good and poor prognosis groups, independent of tumor type (LUAD or LUSC), based on their CAF composition and found that lower patient survival was strongly associated with tumor-like CAFs. In contrast, inflammatory CAFs and interferon-response CAFs were associated with inflamed tumor microenvironments and were linked to a favorable prognosis [117]. The interaction between LUSC cells and CAFs is complex and bidirectional. LUSC cells can activate CAFs, inducing them to adopt a pro-tumorigenic phenotype characterized by increased secretion of factors that support tumor growth. Reversely, CAFs can promote LUSC cell proliferation and invasion, particularly through the PI3K-Akt pathway [116], thereby maintaining the high-risk CAF phenotype. Moreover, the study highlights the potential of the PI3K-Akt signaling inhibitors treatment for LUSC patients [133]. Zhang et al. constructed a CAF prognostic signature model using both scRNA-seq data and bulk RNA-seq data. In this study, three CAF categories were found: iCAFs (inflammatory CAFs), mCAFs (myofibroblast-like CAFs), and apCAFs (antigen-presenting CAFs), which have a role in prognosis and immunotherapeutic response for LUSC. Furthermore, a CAF prognostic signature has been identified that influences critical tumor-promoting pathways, including angiogenesis, EMT, and cell cycle alterations. This signature provides a refined prognosis and may inform immunotherapy strategies, potentially enhancing outcomes in LUSC patients [87]. A recent study identified miR-369 as a key prognostic marker and therapeutic target following the treatment of LUSC cells with CAFs-derived extracellular vesicles (CAFs-EVs). Furthermore, the upregulation of miR-369 stimulated the mitogen-activated protein kinase signaling pathway, controlling migration, invasion and tumorigenesis [47]. CAFs inhibited the activity of high SOX2 levels in a 3D coculture system of LUSC epithelial cells with CAFs and ECM, suggesting that the interaction between the TME and tumor cells plays a key role in LUSC carcinogenesis and in developing new therapeutic strategies [116]. Thus, in order to investigate the molecular biological mechanisms by which CAFs promote LUSC, a recent study identified COL10A1, a member of the collagen family, that encodes collagen type X alpha 1, secreted by CAFs. Furthermore, COL10A1 enhances LUSC cell proliferation and inhibits apoptosis induced by oxidative stress. This effect is facilitated through elevated COL10A1 expression, driven by METTL3-mediated mRNA m6A modification, which ultimately promotes accelerated tumor growth [42].

CAFs are hence essential components of the TME in LUSC, and their involvement in ECM and therapeutic resistance makes them potential therapeutic targets. Understanding the distinct subtypes of CAFs and their interactions with tumor cells is crucial for developing effective treatment strategies, including immunotherapy. Also, targeting CAFs or their associated signaling pathways (tumor angiogenesis, EMT, and cell cycle alterations) may represent a promising approach to inhibit tumor progression and enhance the efficacy of existing treatment modalities, including immunotherapy and targeted therapy [87, 116].

### TME and immunotherapy response in LUSC

LUSC treatment has been greatly advanced by immune checkpoint blockade (ICB) therapy [134]; however some patients do not respond to such treatment, and hence understanding the molecular characteristics of immunosuppressive TME is essential to identify LUSC patients with ICB resistance [135]. Thus, a recent study identified a novel immunosuppressive class of LUSC defined as an exhausted immune class (EIC) with high levels of PDL-1 and IDO protein expression but potential resistance to ICB therapy [135]. The primary immunotherapy strategy for patients with advanced or metastatic NSCLC without driver mutations is represented by ICIs in mono-or combined therapy [136]. A propensity score matching analysis on 832 NSCLC patients found a longer overall survival for patients who received ICI plus chemotherapy after first-line platinum-based chemotherapy compared with those who received ICI monotherapy [137].

A recent meta-analysis revealed that the outcome of NSCLC patients improved with therapy using various PD-1/PD-L1 inhibitors, particularly tislelizumab, pembrolizumab, and nivolumab, which enhance the TME’s ability to resist tumor cell growth [138]. However, while these inhibitors show promising efficacy, their combination with chemotherapy has been associated with a higher incidence of severe adverse effects [138], highlighting the need for strategies that balance therapeutic benefits with manageable toxicity.

Targeted therapies such as epidermal growth factor receptor (EGFR) and KRAS inhibitors have proven to be beneficial only for a reduced number of LUSC patients [139, 140]. A panel of 6 genes (BHMT2, FES, HSPB7, NOVA2, LPAP2, and SEMA3B) was identified as potential biomarkers of TME-related genes based on immune and stromal scores of LUSC patients of TCGA and validated on additional two data sets (GSE4573 and GSE17710) [94]. High immune risk LUSC patients exhibit a higher presence of immunosuppressive M0 macrophages. These macrophages contribute to an immunosuppressive TME, which can hinder the body’s immune response to cancer and affect the efficacy of immunotherapies [84].

Single-cell RNA sequencing analysis of LUSC patients identified a novel immunosuppressive receptor (TIGIT) Tregs and exhausted CD8 + T cells [140], indicating that upregulation of TIGIT might stimulate the development of an immunosuppressive microenvironment and suppress the cytotoxic capacity of CD8 + T cells. This is relevant because targeting TIGIT could help restore anti-tumor immunity and enhance the efficacy of immunotherapy in LUSC. Furthermore, the construction of SPP1 + macrophage-based regulatory networks demonstrated a potential therapeutic target for modulating the TME and improving treatment responses in LUSC [141].

Myeloid cells have been identified as actively involved in tumor growth, angiogenesis, and metastasis in various types of cancer [142]. Myeloid-derived suppressor cells (MDSCs) are a heterogeneous population of cells comprising monocytic (M)-MDSCs, polymorphonuclear (PMN)- MDSCs, and immature myeloid cells [84], which are major regulators of immune responses in various diseases, including cancer [143]. MDSCs contribute to tumor growth through TME remodeling [126, 144] and play an important role in the direct inhibition of T cells but also promote the development and maturation of Tregs, TAMs, and CAFs by generating an immunosuppressive network [145–147]. The frequency of MDSCs was found to significantly increased in LUSC patients [148] participating in the immune escape of LUSC and in developing novel therapeutic strategies for this type of cancer [148]. MDSCs have been involved in resistance to anticancer therapies and are associated with an inhibitory effect of chemotherapy on the immune system. Thus, circulating CD14 + S100A9+ cells have been associated with poor response to cisplatin and other chemotherapeutics in NSCLC patients [149, 150].

TAMs play a crucial role in tumor progression, serving as key components of the TME [69, 151, 152]. TAMs were demonstrated to induce EMT via the TGF-β/Smad/ZEB pathway in LUSC cells [121], meaning that this mechanism contributes to enhanced tumor invasiveness, metastasis, and therapy resistance LUSC. By promoting EMT, TAMs facilitate the acquisition of a more aggressive phenotype in cancer cells, which may lead to poor prognosis and reduced treatment efficacy. Understanding this interaction highlights the potential of targeting TAM-mediated signaling pathways as a therapeutic strategy to counteract tumor progression in LUSC.

In high-risk LUSC patients, TAM targets were significantly upregulated, including CD47, CD73, SIRPA, and TIM-3, which correlate positively with the immune risk score [84]. This suggests that the poorer prognosis in these patients is partly attributed to an immunosuppressive microenvironment.

Furthermore, natural killer (NK) cells are cytotoxic innate-like lymphocytes that identify and eliminate tumor cells through their capacity to release immune-stimulating cytokines [153, 154]. Villegas et al. conducted a study using cytofluorometric methods on samples from fifty patients with primary LUSC to evaluate the role of tumor infiltrating natural killer cells subset CD57 (TINK). Interesting, this was the first study that confirmed a significant direct correlation between of TINK cells and time survival has been found, enhancing the need to develop new therapeutic strategies destinated to increase NK cell activities in these patients [122]. On the other hand, dendritic cells (DCs) are key components of TME that promote antitumor T-cell responses and activate naïve T cells [155, 156]. The function of glypican-3 (GPC3) as a potential new candidate for LUSC patients immunotherapy was confirmed by the finding of an increase in progression, migration and invasion of LUSC cells. The regulation of GPC3 is related to the cell cycle and the PI3K/AKT signaling pathway [124].

Overall, the interplay between the TME and cancer cells involves various types of cells, which are currently not fully understood. Hence, we can see that the interplay between the TME and cancer cells involves various types of cells, which are currently not fully understood.

### Hypoxia and angiogenesis drive drug resistance in LUSC

Hypoxia is another key feature of the TME, arising from insufficient oxygen supply due to rapid tumor growth and abnormal vasculature [108]. This low-oxygen environment triggers the activation of hypoxia-inducible factors (HIFs), which drive the expression of genes associated with survival under adverse conditions, including those involved in angiogenesis, metabolic reprogramming, and resistance to apoptosis [157–159]. In hypoxic conditions, modifications in the biology of stromal cells within the TME are stimulated through mediators of transcriptional hypoxic responses, including HIF1α and HIF2α. These mediators promote gene transcription, producing hypoxic and stromal responses that promote angiogenesis [160, 161]. The development of abnormal angiogenesis and hypoxia in the TME stimulates tumor development and resistance to therapy [162, 163]. Furthermore, TAMs, with immunosuppressive effects, can be enrolled by tumor-hypoxic areas that can suppress the activation of immune cells, e.g. T cells [164, 165].

In LUSC, hypoxia-induced HIF activation triggers EMT, enabling cells to detach and migrate [166]. Additionally, hypoxia stimulates angiogenesis through VEGF, reshapes metabolism, and facilitates immune evasion, collectively supporting metastasis [52, 167]. These adaptations highlight hypoxia as a crucial precursor to metastasis and a potential target for therapeutic intervention in advanced cancers [168].

In a study using LUSC and LUAD cell lines cultured under both normoxic and hypoxic conditions, a significant increase in the proportion of ALDHhi cells was observed in LUSC under hypoxia, suggesting an enrichment of cancer stem-like cells. This effect was mediated through the Wnt/β-catenin pathway, highlighting its role in maintaining stemness and therapy resistance in NSCLC. Hence, targeting both hypoxia and the Wnt/β-catenin pathway can be considered as a strategy to overcome resistance and improve treatment outcomes for NSCLC patients [169].

Wu et al. constructed and validated a hypoxia-related model for LUSC through which gemcitabine exhibited potential sensitivity and selectivity for patients with a low risk of hypoxia 3 genes (HELLS, GPRIN1, and FAM83A) have been identified in this study associated with hypoxia, having a great potential to be used as targeted therapy for hypoxic LUSC patients. Furthermore, the study’s results showed a correlation between hypoxia and the immune microenvironment of LUSC [170]. Hence, this study underscores the critical role of hypoxia in shaping the tumor immune microenvironment of LUSC and highlights potential therapeutic targets for hypoxic tumors. The identification of these hypoxia-associated genes suggests novel avenues for precision medicine approaches, where targeting these genes could improve treatment outcomes.

### CSCs-subpopulation of tumor cells-mediated drug resistance in LUSC

CSCs are a subpopulation of tumor cells that can initiate and sustain tumor growth, as well as drive metastasis and recurrence [63, 135]. As a subpopulation of tumor cells with self-renewal capacity and the ability to drive tumor heterogeneity, CSCs are supported by the TME, which sustains their renewal and contributes to angiogenesis, immune remodeling, and tumor invasion [58]. By adapting to hypoxia and inflammatory signaling, CSCs enhance their metastatic potential and develop resistance to therapy, contributing to poor treatment outcomes [171–173].

CSCs can activate DNA repair mechanisms, protect from ROS action, and reactivate the drug efflux system, thus stimulating tumor drug resistance [174]. Specific cell surface markers, such as CD133, c-KIT, and ALDH1A1, identified the tumor cell population with stem cell characteristics for NSCLC studies [175, 176]. These markers are crucial not only for isolating and characterizing CSCs but also for developing targeted therapies that aim to eliminate these highly resistant cells, ultimately improving treatment efficacy.

Guo et al. found, in a recent study, that CSCs isolated from LUSC are resistant to cisplatin [59]. This finding is particularly significant given that cisplatin-based chemotherapy remains a standard treatment for LUSC. The intrinsic resistance of CSCs suggests that conventional therapies may be insufficient to eradicate the tumor, highlighting the urgent need for novel therapeutic strategies targeting CSC-specific survival mechanisms to improve treatment outcomes [59].

Heterogeneous regulation of distinct CSC subpopulations has been identified in both LUSC and LUAD, with sustained hypoxia selectively increasing EpCAM expression in LUSC [170]. In the same study, SOX2 expression was downregulated by targeting ITGB4 in SOX2-expressing CSCs, sensitizing the cells to cisplatin. Furthermore, the synergistic effect of carfilzomib and cisplatin inhibited CSC proliferation by downregulating the expression of ITGB4 and SOX2 [59]. SOX was overexpressed in various types of cancer and stimulates tumorigenesis by promoting proliferation, stemness features and metastasis [177]. The SOX family develops a suppressive TIME by recruiting suppressive immune cells, increasing the secretion of immune inhibitory molecules and suppressive cytokines, leading to immunotherapy resistance in many patients [177]. In LUSC cancer cells, SOX2 recruits tumor-associated neutrophils and promotes the secretion of CXCL5, thereby increasing the progression of lung cancer cells and influencing the TME [178]. CAFs decrease the expression of SOX2 to prevent dysplasia; overexpression of SOX2 is associated with the transition from hyperplasia to dysplasia in lung cancer [116]. Recent studies have demonstrated that SOX2 is overexpressed in human LUSC compared to adenocarcinomas [116], with a detection rate of 20–65% in LUSC and 6–20% in LUAD [179, 180].

Furthermore, in vivo studies demonstrated that SOX2 overexpression can occur early during LUSC carcinogenesis and can be lost during cancer progression [181]. Upregulation of SOX2 was positively correlated with drug resistance and poor survival of cancer patients [182]. CSCs play a crucial role in drug resistance, driven by their adaptability within the TME and mechanisms like SOX2 overexpression, which promote tumor survival, metastasis, and therapy resistance. An integrative network-based analyses have revealed that SOX2, dependent signaling, intersecting with TME–associated pathways, creates vulnerabilities that can be exploited through rational combination therapies, such as co-targeting AKT and mTOR to overcome TME-driven therapeutic resistance [183].

### Combination therapies targeting the TME in LUSC

Combination therapies can be focused on reprogramming the TME, augmenting antitumor immunity, and minimizing resistance [184]. Unlike LUAD, LUSC has a small proportion of recurrent oncogenic driver mutations that can be targeted with conventional tyrosine kinase inhibitors, leading to limited benefit from targeted therapies traditionally available for LUSC [185, 186]. This, in turn, has evolved in a more comprehensive approach for immunosuppressive TME with the use of both immune-modifying agents, TME–oriented approaches (e.g., immune checkpoint inhibitor plus chemotherapy, anti-angiogenic agents, or other immunomodulatory combinations) to overcome the immunosuppressive TME and enhance clinical outcomes in LUSC [187]. LUSC possesses a highly immunosuppressive and heterogeneous TME, which includes immune cells, CAFs, abnormal vasculature, ECM, and soluble mediators including cytokines and chemokines. Furthermore, unlike LUAD, LUSC features rare viable oncogenic drivers, thus enhancing the need for combining strategies focused on the TME [188, 189].

Table 5 summarizes preclinical studies investigating combination therapies targeting TME components in LUSC, These data underscore the predominance of TME-based combination strategies in LUSC therapy, particularly in the absence of actionable oncogenic drivers.

**Table 5:** Preclinical studies investigating TME targets in LUSC

Combination strategy	TME target(s)	Mechanism / rationale	Key evidence	References
ICI + chemotherapy	Tumor cells, immune cells	Chemotherapy induces immunogenic cell death and enhances antigen presentation; ICIs reinvigorate exhausted T cells	Pembrolizumab + platinum-based chemotherapy significantly improves OS and PFS in metastatic squamous NSCLC	[190, 191]
ICI + anti-angiogenic therapy	Abnormal vasculature, hypoxia	Vascular normalization improves immune infiltration and reduces hypoxia-driven immunosuppression	Preclinical and early clinical evidence supports immune–vascular crosstalk in squamous NSCLC	[192]
	Tumor angiogenesis, TME hypoxia	Endostatin remodels tumor vasculature and enhances immune activation when combined with PD-1 blockade	PD-1 inhibitors + chemotherapy ± endostatin improve survival in advanced LUSC	[193]
Dual immune checkpoint blockade (PD-1/PD-L1 + CTLA-4)	T cells	Targets distinct immune inhibitory pathways to enhance T-cell priming and effector function	Dual-checkpoint strategies discussed for squamous NSCLC with TME-driven rationale	[194]
ICI + radiotherapy	Tumor stroma, immune cells	Radiotherapy increases neoantigen release and promotes systemic immune responses (abscopal effect)	Consolidation immunotherapy following radiotherapy improves outcomes in locally advanced NSCLC	[195]

Combination therapy approaches are increasingly utilized for modulation of the TME to improve efficacy in clinical trials of the LUSC population (Table 6). Combination of immune checkpoint inhibitors and chemotherapy induces immunogenic cell death, neoantigen release, and enhanced T-cell infiltration in the TME, thus enhancing antitumor immune responses [187]. As another approach, dual immunotherapy strategies and bispecific agents inhibit multiple immunosuppressive pathways simultaneously (e.g., a combination of PD-1 with TIM-3) in order to block the intricate and redundant immune evasion mechanisms of the LUSC TME [196]. Anti-angiogenic combinations to also normalize aberrant tumor vasculature and alleviate hypoxia are further targeted, enabling a return to immune cell access and reducing immune exclusion [197]. These TME-focused therapeutic strategies represent a shift toward rational combination therapies in LUSC and utilize immune activation and microenvironmental remodeling to overcome resistance and improve clinical outcomes.

**Table 6:** Clinical trials focused on combination therapies targeting the TME in LUSC

Trial ID	Therapy	Pathology	Primary Focus / TME Target	Notes / Status
ORIENT-12 (NCT03629925)	Sintilimab + platinum + gemcitabine	First-line advanced/metastatic squamous NSCLC	PD-1 blockade + chemotherapy to enhance antitumor immune responses	Phase III; chemotheraphy and immunotherapy improves survival outcomes and alters TME immune infiltration.
RATIONALE-307 (NCT03594747)	Tislelizumab + chemotherapy	First-line advanced squamous NSCLC	PD-1 inhibition + chemotherapy, modulating TME immune activity	Phase III; improved progression-free survival vs. chemo alone.
AK112 (NCT04736823)	AK112 (PD-1/VEGF bispecific) + chemotherapy	Advanced squamous NSCLC	Dual targeting immune checkpoints and angiogenesis (TME vasculature)	Phase II; evaluating safety and efficacy of TME-modulating bispecific + chemo.
Camrelizumab + Apatinib (NCT04379739)	PD-1 inhibitor + angiogenesis inhibitor	Locally advanced or unresectable NSCLC, including LUSC	Anti-angiogenic modulation + PD-1 blockade to normalize TME and enhance immunity	Phase II trial showing promising major pathological response.

## Conclusions

The TME has emerged as a key area of research in LUSC, since its composition plays a critical role both in therapeutic reaction against tumors and drug resistance or in disease progression. The LUSC TME encompasses stromal and immune cell populations, hypoxic regions and ECM elements, each of which plays a role in the therapeutic effect of the drug. Cancer-associated fibroblasts and others components associated with the malignant state modulate the ECM by forming a tumor-supportive niche and resistive phenotype, whereas immunosuppressive immune populations, including regulatory T cells and tumor-associated macrophages, hinder this effectivity with all immune therapies. Hypoxia aggravates resistance by leading to reduced sensitivity to radiotherapy and aggressive tumor behaviour, while ECM modification hinders drug penetration and tumor progression. Approaching these TME features is thus a potential strategy to improve treatment efficacy in LUSC. There is a scope for drug delivery and therapeutic sensitivity improvements through ECM remodeling, stromal cell modulation, immune reprogramming and hypoxia attenuation. By overcoming the specific microenvironmental constraints inherent in LUSC and in particular in absence of actionable oncogenic drivers, TME-directed strategies offer a rational pathway to more effective and resilient therapeutic approaches, which emphasizes the importance of research on TME-targeted approaches.

## Data Availability

Not applicable.

## Abbreviations

LUSC: Lung squamous cell carcinoma
TME: Tumor microenvironment
ECM: Extracellular matrix
LUAD: Lung adenocarcinoma
CAFs: Cancer-associated fibroblasts
TAMs: Tumor-associated macrophages
ROS: Reactive oxygen species
CSCs: Cancer stem cells
EMT: Epithelial-to-mesenchymal transition
ICI: Immune checkpoint inhibitor
DCs: Dendritic cells
GPC3: Glypican-3
Tregs: Regulatory T cells
MMPs: Matrix metalloproteinases
SMA: Smooth muscle actin
ICB: Immune checkpoint blockade
HIFs: Hypoxia-inducible factors
